# Long-term observations reveal the formation process of branching systems of the genus *Sasa* in Bambusoideae

**DOI:** 10.1093/aobpla/plaa054

**Published:** 2020-09-23

**Authors:** Hiroko (Kawabata) Niimiya, Kazushige Kawabata

**Affiliations:** 1 Faculty of Advanced Life Sciences, Hokkaido University, Sapporo 060-0810, Japan; 2 Executive Administration Office, Niigata University, Niigata 950-2181, Japan

**Keywords:** Architecture, Bambsoideae, branching system, bud fate, genus *Sasa*, internode length, snow accumulation

## Abstract

Clarifying the endogenous processes that construct gross aerial shapes such as branching architecture in plants is crucial to understanding how branching contributes to plant adaptation to environments. Architectural analysis is powerful in decomposing the branching process, by comparing observations of plant growth among closely related taxa. The genus *Sasa* (Gramineae: Bambusoideae) contains three major sections *Crassinodi*, *Sasa* and *Macrochlamys.* These sections exhibit characteristic branching architectures and are distributed separately across the Japanese archipelago, in relation to macroclimatic conditions such as snow accumulation. Our study aimed to quantitatively reveal the endogenous processes underlying branching architectures in the three sections of *Sasa*. Long-term observations were carried out branch architectural development on Hokkaido Island from 1979 to 2012, which corresponded to the flowering interval of the genus. The results revealed that the three characteristic branching systems of the genus arise mainly from four endogenous processes (distribution of lateral buds on a culm, internode length arrangement along a culm, determination of the fate of lateral buds, development of branching with culm fragility due to ageing) and their interactions with environmental conditions, especially snow accumulation. These processes are coordinated with each other over the life span of a single shoot in developing branching architecture.

## Teaser

The genus *Sasa* (Gramineae: Bambusoideae) contains three major sections. These sections exhibit characteristic branching architectures and are distributed separately across the Japanese archipelago, corresponding to snow accumulation. Long-term observations were carried out quantitatively on the development of the architectures over Hokkaido Island in the period >30 years, corresponding to the flowering interval. The results revealed that the three characteristic branching systems develop mainly through four endogenous processes and in response to the snow accumulation, distribution of lateral buds on a culm, internode length arrangement, determination of the fate of lateral buds and development of branching with culm fragility.

## Introduction

Shoots of spermatophytes form ramifying systems. Branching architecture, that is, the coarse aerial shape of plants, develops as plants age and/or in response to environmental changes. Alterations of this process lead to diversification or evolution of branching architecture. Architectural analyses are a powerful tool to decompose the process and to reveal endogenous processes that give rise to branching architecture (e.g. Barthélémy and Caraglio 2007). This approach enables us to connect macroscopic features of branching morphology with cellular-level processes (e.g. Bowman *et al.* 1991; Coen and Meyerowitz 1991).

The genus *Sasa* (Gramineae: Bambusoideae) is suitable for this approach, because several closely related taxa in the genus develop distinct and characteristic branching architectures and occur in different climatic regions. The phylogenetic relationships among these taxa are problematic, however, despite many studies ranging from macroscopic observations to molecular analysis (e.g. Miyabe 1890; Kobayashi and Furumoto 2004). The reasons for this include the fact that *Sasa* typically disperses clonally and has organs that vary largely in size, it hybridizes easily, even with other genera (Muramatsu 1994) and it has a long flowering interval. Furthermore, elements of branching architecture associated with taxonomic distinctions have not been elucidated in enough detail to reveal the underlying endogenous processes (e.g. Nakai 1931, 1935; S. Suzuki 1978): for example, section *Crassinodi* is considered to exhibit a small amount of branching near the base; section *Sasa* shows scattered branching from the base to the upper culm; and section *Macrochlamys* has dense branching on the upper culm.

**Figure 1.**
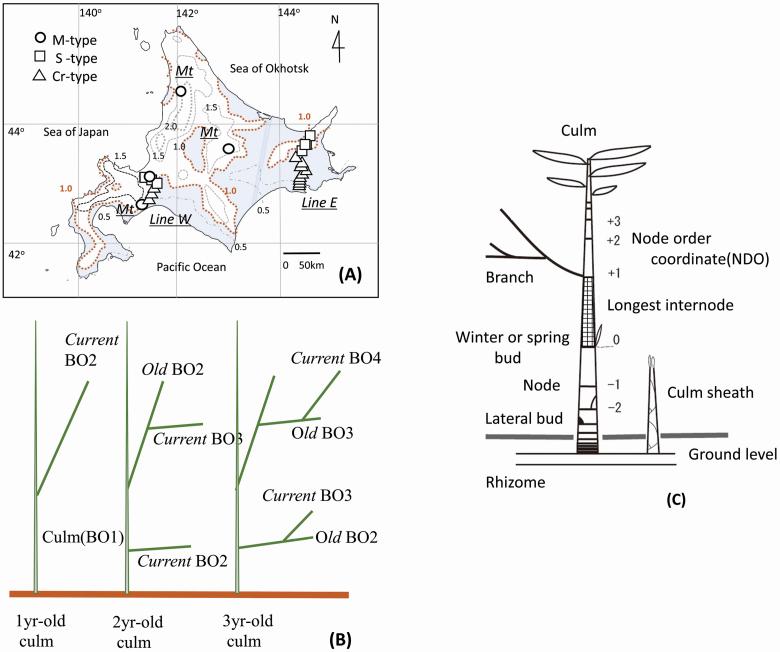
(A) Location of 23 experimental plots in Hokkaido Island, assigned to the Cr-, S- and M-types of the ecological functional unit in the genus *Sasa*. *Line W*: between the Pacific Ocean and the Sea of Japan, *Line E*: between the Pacific Ocean and the Sea of Okhotsk, *Mt*: the highland regions, dotted lines: averaged maximum snow accumulation (1941–70) (in metre) and shaded areas: <l m depth. (B) The branching order (BO) of the branched system. See the text in Materials and Methods. (C) Schematic drawing of culms with node order coordinates (NDO) **[see**  **Supporting Information—E2****]**.

In our previous studies, we applied a grouping approach from an ecological viewpoint (e.g. Botkin 1975), and classified culm assemblages of the genus into three ecological functional units excluding intermediate and composite ones in Hokkaido Island. These culm assemblages are defined in terms of morphological characteristics, ecological behaviours and distribution areas. These units were named as the Cr-, S- and M-types, which were substantially assigned to two sections and the representative species of another section: sections *Crassinodi* and *Sasa*, and *Sasa kurilensis* in section *Macrochlamys*, respectively (Ito and Niimiya 1982–1983; Kawabata (Niimiya) and Ito 1992; Ito and Kawabata (Niimiya) 1998). These distribution areas were largely distinct from each other, though the three sections were continuously distributed across 90 % of the island’s forest understorey area. The boundary between the Cr-type and the other two types, S-and M-types, corresponds to an average maximum snow depth of 1 m, as drawn in Fig. 1A (Sapporo District Meteorological Observatory 1973): the Cr-type lies on the Pacific Ocean side of the island, which receives little snowfall, while the S- and M-types lie on the Sea of Japan side of the island, which experiences heavy snow accumulation.

The genus *Sasa* exhibits different life-forms, corresponding to differences in winter precipitation across the Japanese archipelago (Suzuki 1962*a*). These life-forms were categorized according to Raunkiær’s scheme, based on the positions of branching and winter bud formation: hemicryptophyte for section *Crassinodi*, and chamaephyte for sections *Sasa* and *Macrochlamys* (Suzuki 1961, 1962*b*, 1978; Usui 1961; Yamazaki *et al.* 1974). Such morphological diversification correlated with the two regions was also observed in the genera of other families across the archipelago, such as *Artemisia*, *Cacalia* and *Gentiana* for organ size, and *Camellia*, *Aucuba*, *Ilex*, *Cephalotaxus* and *Daphniphyllum* for growth habit (e.g. Yamazaki 1959; Fukuoka 1966; Hotta 1974). This morphological variation exists, however, only between species and subspecies or ecotypes in these genera. It should be noted that the genus *Sasa* contains many species and spans a much larger area than Hokkaido Island.

The objective of the present study is to reveal the endogenous processes that give rise to branching architecture in the Cr-, S- and M-types of the genus *Sasa* in Hokkaido, using quantitative architectural analysis. Clarifying these processes is crucial to understanding not only branching development itself, but also how branching adapts to differing environments and leads to evolutionary diversification.

Branching in *Sasa* develops in the following simple ways. Shoots (either culms or branches) elongate each year through intercalary growth, without secondary growth or thickening, in common to genera of Bambusoideae. Only one lateral bud is produced per node, such that the branching order increases by one each year as the culm ages (Fig. 1B) (Makino and Shibata 1901; Muroi 1937, 1969; Ueda 1956; Usui 1957; McClure 1966). The genus reproduces asexually by rhizome for many decades until flowering, after which the plant dies—as also occurs in other temperate genera of Bambusoideae. The branching architectures are built repeatedly in the vegetative stage during the period.

We carried out long-term observations of branching architecture quantitatively, noting the following parameters: distribution of lateral buds on a culm, internode length arrangement along a culm, determination of the fate of lateral buds and development of branching with culm fragility due to ageing. Our objective was to reveal the endogenous processes that give rise to distinct branching patterns in the three subtypes of *Sasa* in the different environments, with particular focus on snow accumulation. We confirmed the long-term stability of branching formation processes during the life of individual plants, and also in relation to environmental changes during the 33-year experimental period (1979–2012), which coincided with an interval of synchronous flowering (Mohri 1976; Nishikoori 2007).

## Materials and Methods

### Sample collection

The genus *Sasa* (Gramineae: Bambusoideae) occurs in the Japanese archipelago and neighbouring regions, forming the most northern distribution area of the Bambusoideae.

The Bambusoideae consists mostly of temperate genera in the archipelago. A major group within the temperate genera, the *Sasa* group, is classified by a persistent leaf sheath around aged leaf blades, called the ‘culm sheath’ (Muroi 1937). The genera of this group are distributed in more northern regions than those with no culm sheath. The genus *Sasa* belongs to the *Sasa* group and is distributed widely, dominating forest understorey and making *Sasa* grasslands in diverse habitats including windswept cliffs above treeline, marshland and coastal dunes across the archipelago.

The examined materials were aerial culms of the three sections (*Crassinodi*, *Sasa* and *Macrochlamys*) in Hokkaido Island, where the *Sasa* group occurs alone and the genus *Sasa* is most abundant (e.g. Toyooka *et al.* 1983). Twenty-three experimental plots (ca. 0.25 ha per plot) were set in the central and eastern parts of the Island, which are characterized by intermediate conifer/deciduous broad-leaved mixed forests, with subalpine forests at higher elevations (Tatewaki 1958). Culm assemblages at the plots were previously assigned to the Cr-, S- and M-type ecological functional units. The plots were arranged along geographical gradient lines between the Pacific Ocean and the Sea of Japan (*Line W*), or between the Sea of Okhotsk and the Pacific Ocean (*Line E*) and the highland regions of Hokkaido (*Mt*) denoted in Fig. 1A and Table 1  **[see**  **Supporting Information—E1****]**.

**Table 1.** The examined number of culms and their assemblages at the 23 experimental plots, ecological functional unit type of the genus, line, label, coordinates, altitude, snow accumulation (measured in February 1981 at the Cr- and S-type plots and in February 2012 at the M-type plots; in parenthesis mean values were from the climate data of Japan Meteorological Agency between 1981 and 2010 at the nearest observatory) and canopy species (detailed information is given in **Supporting Information—E1**). The experimental plot M1-S was arranged neighbouring to M1 and under the harsh condition where the culms became much short.

Type	Line	Experimental plot label	Coordinates	Altitude (m)	Snow accumulation (mm)	Canopy species	Number of assemblages	Number of culms
Cr	E	E1	43°3′N, 144°28′E	15	184 (260)	Qm, Cc, Jm	7	168
Cr	E	E2	43°6′N, 144°29′E	17	160 (260)	Qm, Acm, Pha	6	160
Cr	E	E3	43°9′N, 144°30′E	10	170 (–)	Alj, Bp, Acm, Qm, Fm	6	175
Cr	E	E4	43°12′N, 144°30′E	18	137 (–)	Qm, Soc	7	184
Cr	E	E5	43°14′N, 144°33′E	27	300 (–)	Bp, Ud, Acm, Lk	7	185
Cr	E	E6	43°18′N, 144°37′E	54	200 (580)	Bp, Alh, Sas, Lk	6	189
Cr	E	E7	43°23′N, 144°33′E	61	190 (–)	Lk	7	228
Cr	E	E8	43°26′N, 144°32′E	115	550 (–)	Lk, Fl	7	241
Cr	E	E9	43°30′N, 144°28′E	114	440 (–)	Sah, Bp, Qm, Ma	8	221
Cr	W	W1	42°41′N, 141°42′E	2	170 (280)	Qm, Alj, Acm, Tj	5	238
Cr	W	W2	42°44′N, 141°47′E	10	250 (660)	Lk	5	212
Cr	W	W3	42°40′N, 141°37′E	22	390 (–)	Qm, Tj, Acm	10	400
S	E	E10	43°43′N, 144°30′E	154	720 (900)	Tj, Ud, Bp, Lk	8	239
S	E	E11	43°47′N, 144°32′E	59	560 (900)	Bp, Qm, Tj, Sas, Lk	8	234
S	E	E12	43°47′N, 144°33′E	60	0 (900)	Qm, Ud, Acm, Ma, Kp, Soc	8	157
S	E	E13	43°51′N, 144°34′E	25	540 (960)	Fm, Alj, Ud	8	262
S	W	W4	43° 0′N, 141°47′E	35	1020 (–)	Open	5	192
S	W	W6	43°3′N, 141°30′E	55	1400 (–)	Pij, Oj, Tj	7	193
S	W	W7	43°1′N, 141°20′E	12	930 (970)	Tj, Ud, Acm	7	306
M	W	W5	43°1′N, 141°33′E	41	1060 (–)	Abs, Oj, Acm	12	124
M	Mt	M1	44°57′N, 142°8′E	413	1620 (1820)	Pig, Abs, Acm	12	191
M	Mt	M1-S	44°57′N, 142°8′E	413	650 (1820)	Open	5	52
M	Mt	M2	43°53′N, 143°1′E	858	2100 (1210)	Abs, Pij	7	65
M	Mt	M3	42°44′N, 141°20′E	277	1450 (–)	Abs, Pij, Tj	7	160
						Total number	175	4776

Independent aerial culms were sampled randomly at the plots from 1980 to 2012. A cutting position of the culms was chosen according to the winter bud distribution on each culm. These culms were assembled in the growing year from 1979 to 2012. Sampling was made without consideration of underground structures linking culms, such as rhizomes. We considered the collected aerial culm as the main stem because it is the first-order axis developing the branching architecture above the ground.

**Figure 2.**
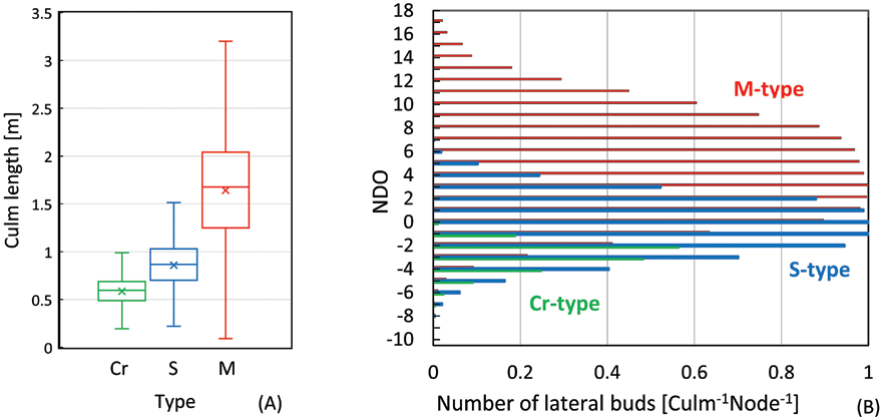
(A) Variation of culm length of all samples without culm broken in the three types using the box plots. The values indicate minimum, lower quartile, median, upper quartile and maximum with mean values of the culm length (*n* = 1696, 838 and 477 for the Cr-, S- and M-types, respectively). (B) The proportion of the number of nodes where a lateral bud was attached to that of all examined nodes at node positions in the NDO, as the number of the bud for a node of a culm for the three types (*n* = 1844, 1036 and 476 for the Cr-, S- and M-types, respectively).

Collected culms were measured when fresh or after drying for 48 h at 60 °C. Box plots show a variation of unbroken culm length of the examined samples (1696, 838 and 477 culms for the Cr-, S- and M-types, respectively) in Fig. 2A; the numbers on the box plots indicate minimum, lower quartile, median, upper quartile and maximum with mean values of the culm length. The mean values with 95 % confidence interval (CI) for all samples were calculated as 0.601 ± 0.007 m for the Cr-type; 0.859 ± 0.018 m for the S-type; 1.663 ± 0.060 m for the M-type.

### Measurements

We measured culm length and estimated the distribution of lateral buds, internode length arrangement, determination of bud fate and development of branching with culm fragility due to ageing. The node order coordinate (NDO) was used to identify node positions on a culm (Fig. 1B), as the genus *Sasa* develops shoots segmentally in a growing season. This coordinate is a 1D axis with node digits along the culm with the origin at the longest internode. The genus *Sasa* has one longest internode identified clearly in the middle part of the culm, in contrast to other genera of Bambusoideae in the Japanese archipelago (e.g. Nomura 1980). The NDO enabled us to compare the characteristics of bud behaviour among different culms and revealed type-dependent characters depending on the node positions (Kawabata (Niimiya) and Ito 1992), regardless of culm length and its underground conditions **[see**  **Supporting Information—E2****]**.

**Figure 3.**
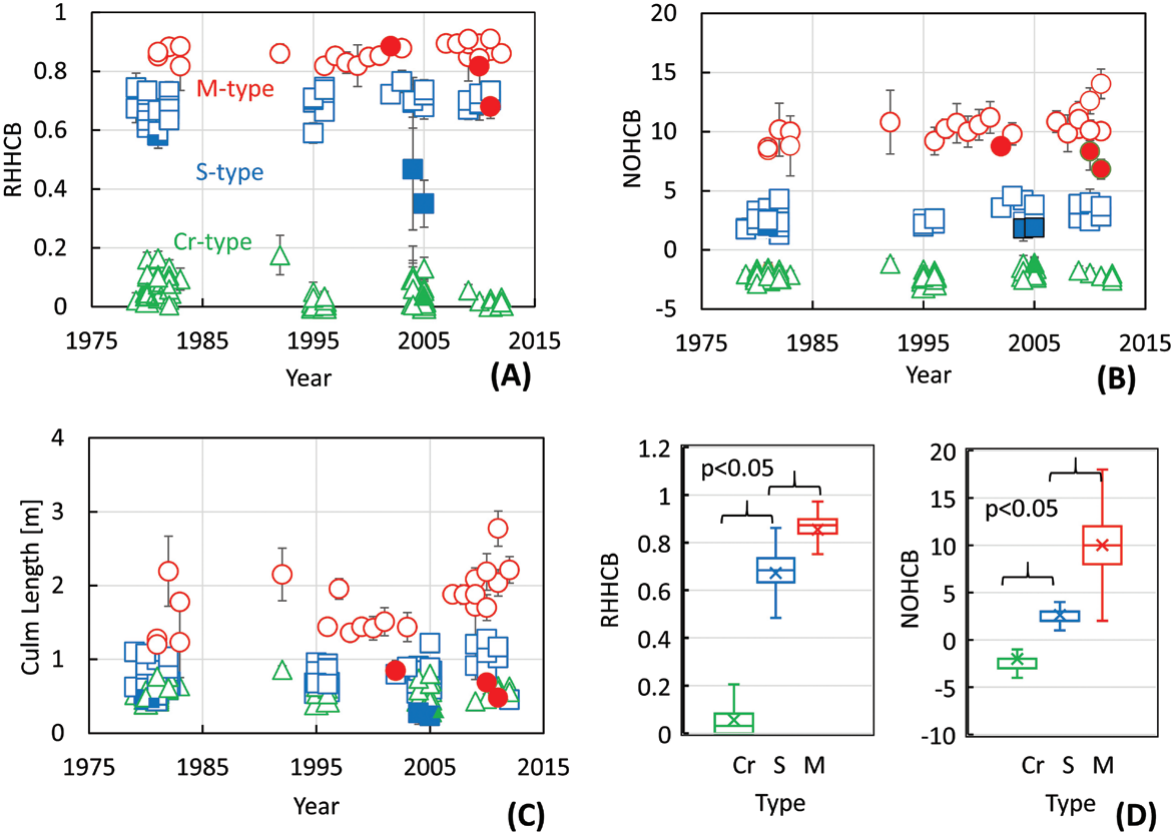
Time dependence of the mean values of highest positions of lateral buds according (A) to relative height (RHHCB) and (B) to node order coordinates (NOHCB) and (C) the values of culm length from 1979 to 2012 with 95% CI. Closed symbols: seven culm assemblages having a mean value of culm length, RHHCB and NOHCB less than half that for all examined samples of the type. (D) The variation of RHHCB and NOHCB of all samples; the numbers on the box plots indicate minimum, lower quartile, median, upper quartile and maximum with mean values of the indices (*n* = 1502, 730 and 351 in A, B, C; *n* = 1696, 838 and 456 for RHHCB in D; *n* = 1693, 838 and 456 for NOHCB in D for the Cr-, S- and M-types, respectively).

The number of lateral buds was calculated at each node position using the ratio of the number of nodes where the bud was attached, to the number of examined culms for culm assemblages for each type. The value of that ratio is the probability or expectation value of a bud occurring at a given node position. The distribution range of lateral buds on single culms can be represented by the highest node position on culm, as shown in Fig. 2C. The following two indices represented the upper limit: relative height of the highest positioned culm bud to culm length (RHHCB), and node order of the node attached to the highest positioned culm bud (NOHCB). The values of RHHCB and NOHCB were averaged for the culm assemblages having more than five culms to be examined (Fig. 3). The total number of the calculated samples was 1502 (74 assemblages), 730 (45 assemblages) and 351(26 assemblages) for the Cr-, S- and M-types, respectively. Box plots show the variation of RHHCB and NOHCB of all samples in the Fig. 3D. The *t*-test on these indices was calculated by statistical analysis software of Microsoft Excel 2016.

**Figure 4.**
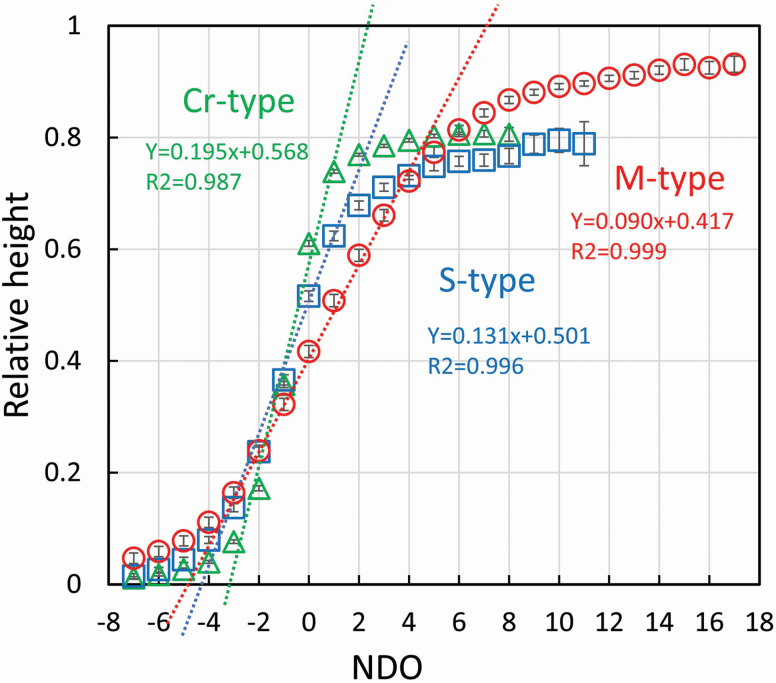
Relative height of node positions to the culm height for the three types using the NDO with 95% CI. The culms were collected between 1980 and 2012. The slopes were obtained by the regression line based on the data between −2 and +1 in the NDO (*n* = 1348, 563 and 303 for the Cr-, S- and M-types, respectively).

Internode length was measured for sample culms having no internodes damaged by insects or animals. The number was 1348, 563 and 303 for the Cr-, S- and M-types, respectively. The distribution of internode lengths along culms was described using the relative height of each node position to the culm height as follows: the value was summed up for each identical node position in the NDO from the base on a culm and the relative height of each node position was calculated to its culm height (culm length) and then averaged for all samples (Fig. 4).

**Figure 5.**
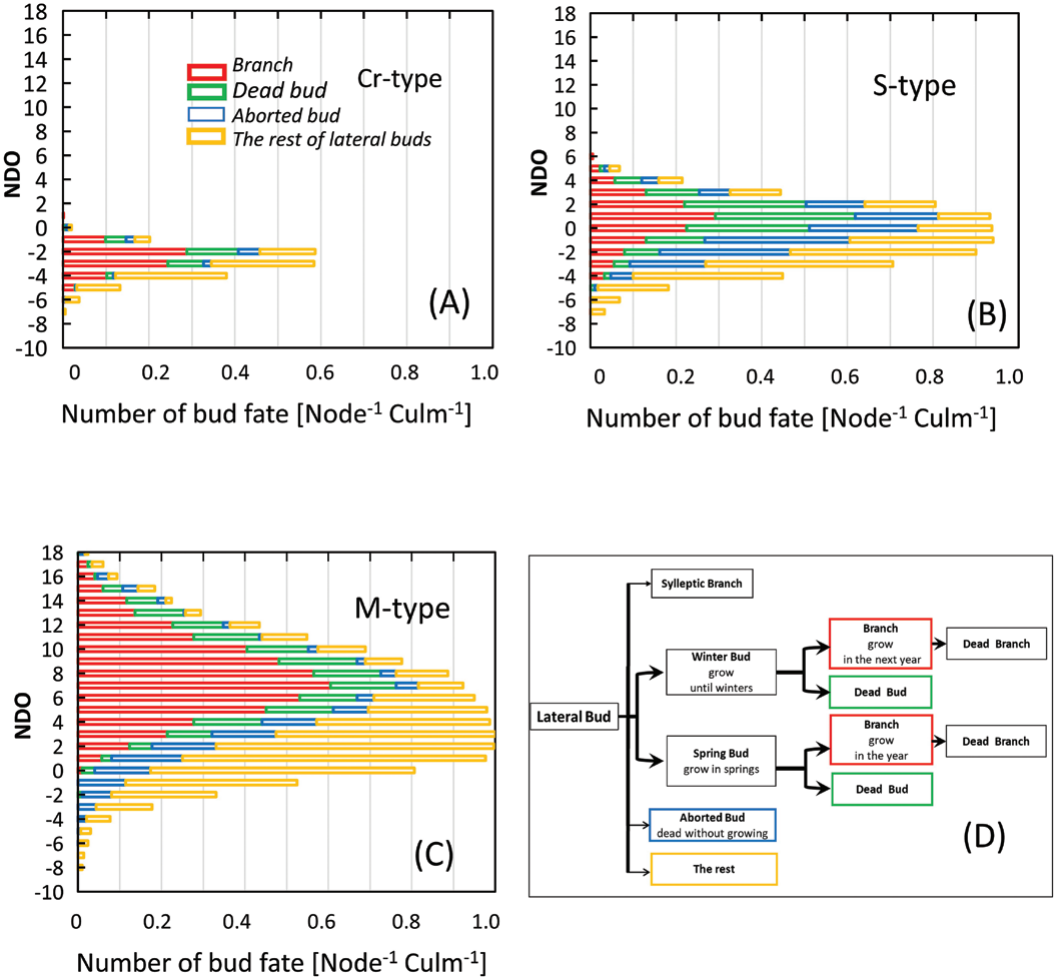
Distribution of the number of four kinds of the fate (Branch, Dead bud, Aborted bud and the rest) of the lateral buds on 1-year-old culms for the three types using the NDO (A, B, C). The ratio of the number of nodes where a lateral bud was attached to that of all examined nodes at node positions as the number of the bud for a node of a culm for the three types. The kinds of bud fate are illustrated (D). The culms were collected between 1980 and 2012 (*n* = 779, 401 and 299 for the Cr-, S- and M-types, respectively).

The fates of buds within 2 years after culm sprouting were observed in the field or estimated for single culms older than 1 year old, and classified into four categories: (i) Branch: lateral buds developed a branch through winter or spring bud, (ii) Dead bud: lateral buds activated and died before branching, (iii) Aborted bud: lateral buds died before growing under culm sheaths or leaf sheaths and (iv) the rest of lateral buds under the sheaths (Fig. 5D). The number was calculated at identical node positions in the NDO (Fig. 5), using the proportion of the number of nodes where the bud was attached to that of the examined culms for culm assemblages. The examined number was 779, 401 and 299 for the Cr-, S- and M-types, respectively.

**Figure 6.**
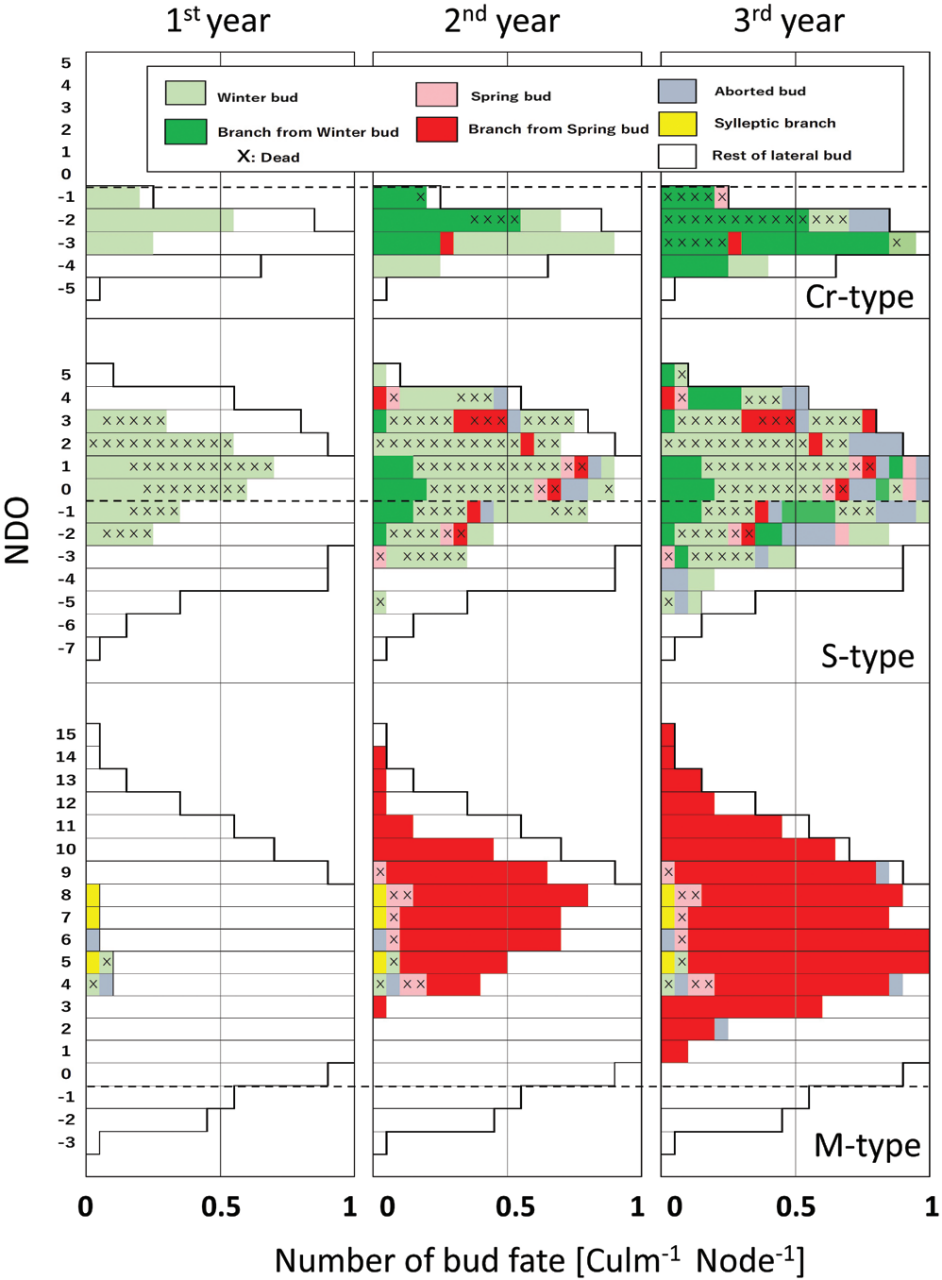
The successive change of bud fate in three consecutive years at identical node positions in the NDO for the three types in the field. The ratio of the number of nodes where a bud was attached to that of all examined nodes at node positions, as the number of the bud fate for a node. The fate was categorized in Fig. 5D.

As lateral buds remain dormant with the capability of growing under culm sheaths or leaf sheaths, the fate of lateral buds was monitored in the field. The bud fate was observed over three consecutive years for the identical culm assemblages at experimental plots W3 (1980–82), W7 (1980–82) and W5 (1994–96) for the Cr-, S- and M-types, respectively (Fig. 6). Three observation years were established: the first year, spring to autumn in the first year of culms sprouting; the second year, winter of first to autumn of the second year; and third year, winter of second to autumn of the third year. Twenty current culms were selected randomly at each quadrat (5 × 5 m^2^ for the Cr-type, 10 × 10 m^2^ for the S- and M-types). The bud fate was recorded one to four times per month in the growing season (April to September) and once or twice in the winter season (October to March). The bud fate was classified into nine categories, as illustrated in Fig. 5D; (i) Sylleptic branch, (ii) Winter bud, (iii) Spring bud: lateral buds grow out of the sheaths in spring, (iv) Branch produced by winter bud, (v) Branch produced by spring bud, (vi) Aborted bud, (vii) the rest of lateral buds, (viii) Dead bud and (ix) Dead branch. The categorized bud fate was described at identical node positions on single culms in the NDO. The number was calculated at identical node positions in the NDO, using the proportion of the number of nodes where the bud was attached to that of 20 examined culms for culm assemblages.

**Figure 7.**
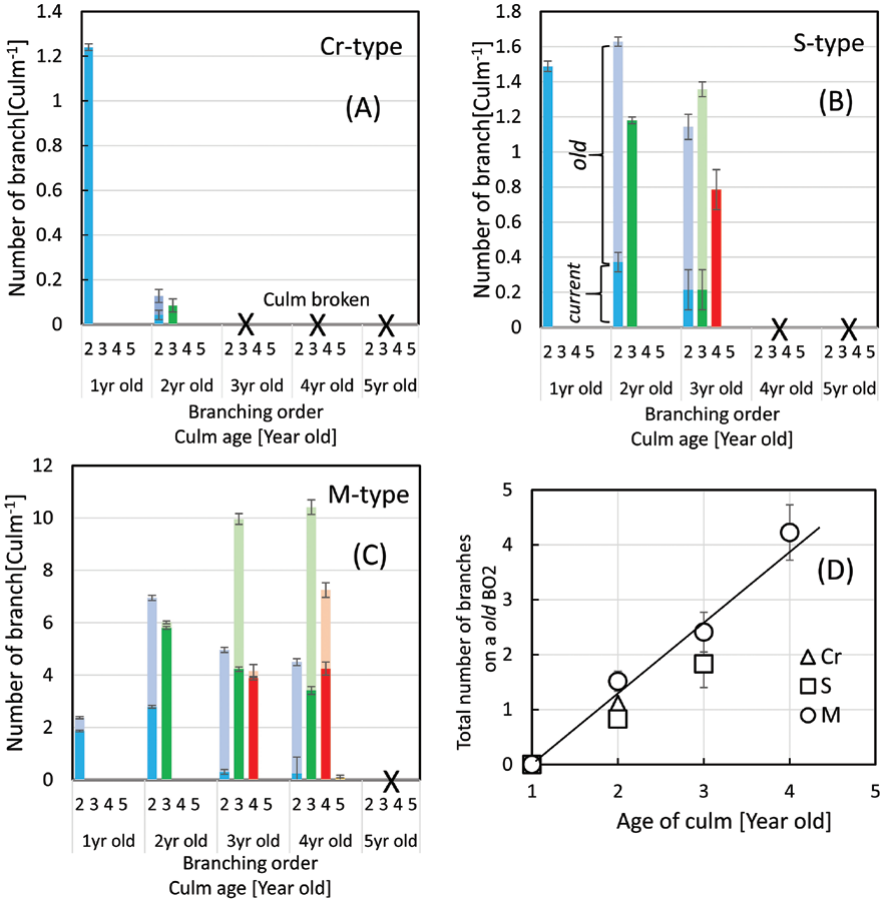
Averaged number (mean ± SE) of branches on single culms plotted as functions of the branching order and the culm age for the three types (A, B, C). The dark and pale solid bars indicate ‘*current*’ and ‘*old*’ branches, and the colours do branching order of branches, as illustrated in Fig. 1C. The total number (mean ± SE) of higher-order branches developed on a single *old* BO2 was plotted as a function of the culm age (D) (*n* = 914, 506 and 243 for the Cr-, S- and M-types, respectively).

The number of branches (mean ± SE) was measured, and the average value was calculated for each degree of branching order on single culms, according to the culm age. The total number of the examined culms was 766, 512 and 243 for the Cr-, S- and M-types, respectively (Fig. 7). The highest degree of branching order generally corresponds to the age of the culm, except for sylleptic branches. The branching order (BO) was named as illustrated in Fig. 1C. One-year-old culms developed branches (BO2) on culms (BO1) and are named ‘*current*’. On 2-year-old culms, *current* BO3 developed on branches (BO2), which are named ‘*old*’, and other *current* BO2 developed on the culms at the same time.

## Results

### Lateral bud distribution on culms

Figure 3 shows the time dependences of the indices of RHHCB, NOHCB and culm length with 95 % CIs (mean ± 95 % CI) for the Cr-, S- and M-types of the genus *Sasa* to confirm long-term stability of lateral bud distribution ranges for each type. These values were also compared with that of culm length to examine the influence of the variation in plant size. Both indices of RHHCB and NOHCB represent the upper limit of lateral bud distribution in the relative height and the NDO, respectively. There was no apparent trend over the experimental period of 33 years, which is comparable to a flowering interval of the genus in Hokkaido Island.

Seven culm assemblages showed less than half of the mean value of the culm length for all examined samples of each type. These culms were expected to be exposed to extremely poor environmental conditions compared with the other culms. The culms grew at a cliff, for example, at the experimental plot E12, where snow accumulation was 0 m despite the S-type region. The RHHCB and NOHCB values of these assemblages, however, belonged to the range depending on types. The results mean that the distribution range of the lateral buds on the culms is little affected by environment. The values of RHHCB and NOHCB (mean ± 95 % CI) were estimated for all samples; 0.0554 ± 0.003, −2.06 ± 0.04 for the Cr-type; 0.672 ± 0.006, 2.62 ± 0.08 for the S-type; and 0.854 ± 0.007, 10.01 ± 0.24 for the M-type, respectively. The box plots for RHHCB and NOHCB are shown in Fig. 3D. The ranges of two indices were separate each other among the three types, while that of culm length overlapped, as shown in Fig. 2A. A *t*-test confirmed that the classification of three types on the indices (RHHCB and NOHCB) was statistically significant at the *P* < 0.05 level. These results indicate that the three types could be classified quantitatively by stable distribution ranges of lateral buds on culms using these indices.

### Arrangement of internode lengths on culms

The branching architecture developed based on node positions arranged on the culms. Figure 4 shows the relative height (mean ± 95 % CI) of each node position to culm length, describing size arrangement of internode length along the culm for the Cr-, S- and M-types. The values were averaged for samples collected over the experimental period; the data were plotted with the identical node positions in the NDO, containing >10 examined culms. The node positions were arranged to look like sigmoid curves for the three types. The NDO dependence of the relative height of the node position was steepest at zero. The relative height at zero and the value of the slope with determination value *R*^2^ was estimated: 0.61 ± 0.005, 0.195 with *R*^2^ = 0.987 (Cr-type); 0.51 ± 0.009, 0.131 with *R*^2^ = 0.996 (S-type); and 0.41 ± 0.01, 0.090 with *R*^2^ = 0.999 (M-type). The slopes were obtained by regression based on data between −2 and 1 in the NDO. The very small CIs suggest that these characteristics remained stable over the long experimental period of 33 years.

### Bud fate on culms

Figure 5 shows fates of buds produced within 2 years after culm sprouting, at identical node positions in the NDO for the Cr-, S- and M-types. The bud fate was classified into four categories, as illustrated in Fig. 5D: Branch, Dead bud, Aborted bud and the rest of the lateral buds.

 The total number of lateral buds over all node positions for a culm was as small as 1.95 per culm for the Cr-type. It was distributed in the narrow range from −7 to +1 in the NDO, as shown in Fig. 5A. Branches of 0.77 per culm were developed from 39.6%. Only 19.2 % of all lateral buds were Dead or Aborted. The total count of lateral buds for the S-type was 6.68 per culm, and the distribution range was wider from −7 to +6 in the NDO, as shown in Fig. 5B. Only 18.7 % of them developed branches of 1.25 per culm, and 44.4 % were Dead or Aborted (3.00 per culm). The total value and the distribution range of lateral buds for the M-type are 13.0 per culm and widest from −8 to +18 in the NDO in Fig. 5C. Their 35.4% developed (4.61 per culm), while 25.0 % were Dead or Aborted. These results show significant differences among the three types, both in the number of lateral buds formed on a single culm and in the ratio of dead and aborted buds to branch establishment.

### Successive bud behaviour on identical alive culms

Figure 6 shows the successive change of bud fate, which was traced on each lateral bud using identical alive culms for 3 years, despite a limited number of examined samples. Bud fate after culm sprouting was classified into nine categories as drawn in Fig. 5D, according to how they changed from one spring to the next: Sylleptic branch, Winter bud, Spring bud, Branch produced by winter and spring bud, Aborted bud, the rest of lateral buds, and Dead bud or branch.

It should be noted that the branches developed through winter or spring from lateral buds differed between types. The total number of lateral buds for the Cr-type was 2.8 per culm in a narrow NDO range from −5 to −1 in the NDO. In the first year, 40.0 % of them became winter buds of 1.0 per culm in the upper range of its distribution. In the second year, all developed branches and 25 % of the branches were broken off at lower nodes after that. One spring bud developed a branch, and winter buds (1.00 per culm) were formed. In the third year, the survived winter buds developed branches (0.80 per culm). All branches of 2 years before were broken off and then remained only 0.85 per culm. The rest of the lateral buds were Dead or Aborted, at the upper distribution range. A few winter buds were formed (0.20 per culm) below these buds, and then 12.5 % of the lateral buds remained as the rest. The result indicates for the Cr-type that a small number of branches developed through winter buds, and they were broken within 2 years.

The S-type culms formed many lateral buds (8.70 per culm) over a wider NDO range from −7 to +5. In the first year, 31.6 % of all lateral buds grew to be winter buds (2.75 per culm), and 78 % of them died. In the second year, surviving winter and spring buds developed branches of 1.15 per culm by winter, half of which developed through spring buds (0.55 per culm). Winter buds (1.65 per cum) were formed by 19.0% of all lateral buds, and 57.6 % of them died. In the third year, both surviving winter buds and one spring bud developed branches of 0.55 per culm. Branches growing through spring buds in the second year had 63.6% of being dead (0.35 per culm), and among examined culms, remaining branches numbered 1.35 per culm. Most of the remaining lateral buds died until the third year at the upper NDO range, while 19.0 % of all lateral buds remained at the lower one. In the second or third years, 13.8% of all lateral buds were classified as Aborted. The result indicates for the S-type that many lateral buds were formed to become both many winter buds and a few spring ones, though most winter buds died. It also shows that a small number of the surviving winter and spring buds, and a large number of buds aborted later, developed into a small number of branches on culms.

The M-type culms formed many lateral buds of 12.7 per culm over culm in the NDO range of −3 to +15. Most of the branches grew through spring buds. There was a small number of Dead and Aborted buds in the first year, except for sylleptic branches. In the second and third year, 59.1 % of all lateral buds grew to be spring buds and developed branches of 7.4 per culm. All branches were retained. The lateral buds remained 37.1% of their number, mainly at a rather lower NDO range under +3. The result indicates for the M-type that many lateral buds were formed and became spring buds, and most of them developed branches without damage in the experimental period.

### Development of branching on culms

The genus *Sasa* develops first- and higher-order branches year by year after finishing culm elongation, as illustrated in Fig. 1B. For the Cr-type (Fig. 7A), 1-year-old culms (BO1) developed *current* BO2 of the number (mean ± SE) of 1.07 ± 0.002 per culm. This value decreased with culm age, and 2-year-old culms develop only a small number of *current* BO2 of 0.043 ± 0.021 per culm. The culms older than the 2-year-old ones were fragile, which distal parts easily broken off. There are no culms with a higher-order branch than BO3. For the S-type (Fig. 7B), 1-year-old culms developed *current* BO2 of 1.49 ± 0.02 per culm. This value decreased steeply with the age of culms, as well as for the Cr-type, and 2-year-old culms developed a small number of *current* BO2 (0.37 ± 0.06 per culm) and *current* BO3 (1.18 ± 0.02 per culm). The values of both *current* BO2 and BO3 decreased to 0.21 ± 0.11 and 0.21 ± 0.11 per culm, respectively, with culm age. The *current* BO4 of 0.79 ± 0.11 per culm was developed on a 3-year-old culm. There are no culms with higher-order branches than BO4 due to its culm being fragile. For the M-type (Fig. 7C), 1- and 2-year-old culms developed *current* BO2 of a large number of 1.87 ± 0.03 and 2.79 ± 0.05 per culm, although the value decreased steeply for 3-year-old ones. Two-year-old culms developed a larger number of *current* BO3 of 5.81 ± 0.05 per culm. These values were kept large as 4.23 ± 0.05 and 3.42 ± 0.15 per culm for 3- and 4-year-old culms. *Current* BO4 developed 3.88 ± 0.06 and 4.25 ± 0.25 per culm on 3- and 4-year-old culms. There are no culms with higher-order branches than BO5 due to its culm being fragile. For all three types within the genus, the number of *current* branches was greatest at the highest order of branching at the culm age, while the culms became fragile and easily broken.

## Discussions

### Formation of branching architecture

The results revealed that four endogenous processes combined to produce the characteristic branching architectures of the three types within the genus *Sasa*: distribution of lateral buds on a culm, internode length arrangement along a culm, determination of the fate of lateral buds and development of branching with culm fragility due to ageing. These four processes enable us to reconstruct the characteristics of the branching architecture for the three types, as follows. (i) The Cr-type culms are short in height and consist of very short internodes except for one or two long ones around the central part. A few lateral buds are formed in a narrow range of culms near the ground level. Most of them develop branches (BO2) through winter bud formation. The culms break easily within 2 or 3 years after sprouting, so that only 1-year-old culms with branches (BO2) are observed in the field. (ii) The S-type culms are taller and have mostly short internodes except for a few rather long ones. Many lateral buds are formed from the ground level up to 70 % of the culm height. Many of these lateral buds become winter buds, and most die. The branches (BO2) develop both through winter and spring bud formation and distribute sparsely on culms. As the culms are broken within 4 years, only 1- or 2-year-old culms developing second- or third-order branches (BO2 and/or BO3) are observed in the field. (iii) The M-type culms are the tallest and have many long internodes around the central part. Many lateral buds are formed from the ground level up to 90 % of the culm height. Many spring buds are formed mainly on the upper part of culms. Few lateral buds die. Many branches (BO2) develop through not winter but spring bud formations. As the culms are tough enough to remain for 5 years, branches from BO2 up to BO5 develop.

**Figure 8.**
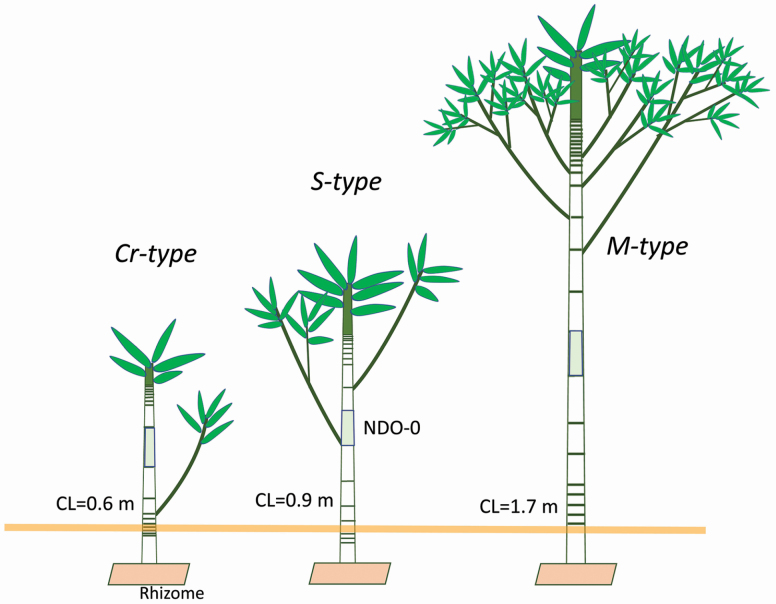
Semi-quantitative description of the branching architecture for the Cr-, S- and M-types in the field, based on the present results.

These results give us a quantitative description of the taxonomic character of the architectures for sections *Crassinodi*, *Sasa* and *Macrochlamys*. Figure 8 shows a semi-quantitative description of the branching architectures for the Cr-, S- and M-types in the field based on the present results.

### Development of branching formation on culms

The total number of higher-order branches that developed on one ‘*old*’ branch (BO2) was plotted as a function of the culm age in Fig. 7D. The number of ‘*current*’ and ‘*old*’ branches with branching order greater than BO3 was summed and divided by the number of BO2 at each culm age. The total number was proportional to culm age, with a common coefficient of 1.38 branches per age among the three types. This result means that all three types of the genus share a similar mechanism to increase branch numbers on old BO2 branches. The branch number continues to increase until the culm (BO1) is broken generally due to fragility by its ageing. In other words, the type-dependent characteristics of branching architectures originate in the distribution of BO2 on single culms and the mechanical fragility of culms.

### Formation of internode length arrangement

The genus develops segmented shoots through intercalary growth, by way of patterns that are partly shared among types in the genus, and partly distinct. In all three types, internode length becomes greatest around the middle part of the culm and becomes shorter with distance from that position. As the flowering interval of the genus is longer than several decades, we usually observed shoots under vegetative growth stage in the field. If flowering should occur, a panicle is formed to be located just above the longest internode, with short rachis internodes at the tip. Similar profiles are observed in the genus *Phyllostachys* in Bambsoideae (Kasahara *et al.* 1984) and the Gramineae grasses such as rice (*Oryza sativa*) (Hoshikawa 1975). This common pattern is considered to be related to long-term vegetative growth resulting from a long flowering interval.

The type-dependent profile is based on the number and length of rather long internodes along a culm. A similar variation was observed among varieties of rice: its tropical varieties with taller culms produce a larger number of long internodes than temperate varieties with shorter culms (Vergara *et al.* 1965; Suetsugu 1968; Inouye 1987). On the other hand, the taller culms represent a larger amount of biomass in the genus *Sasa* (Oshima 1961). Extended growth duration and fertilizer application increase the number and length of long internodes (Takahashi and Takeda 1969; Matsushima 1973; Hoshikawa 1975). Such internode elongation has also been researched from a molecular perspective for rice (Shimizu 1965; Suge and Murakami 1968; Raskin and Kende 1984; Sasaki *et al.* 2002; Azuma *et al.* 2009) and Bamboos (Murakami 1970; Yanagisawa *et al.* 1992). Thus, we consider that the type-dependent internode profiles among the three types are related to their stored nutrients because of a clear difference in their culm height.

### Snow effects on the branching systems

Snow accumulation has opposite effects, depending on snow depth, on branching architecture of plants. Snow covering prevents extreme cold temperatures, dry winds and soil freezing, which cause drying and freezing of the shoots and their winter buds, while the weight of snow can promote branch breakage and its shielding of sunlight can reduce photosynthesis and promote diseases (Tasugi 1929; Shidei 1954; Takahashi 1960; Sakai 1977).

We consider that the endogenous processes underlying branching architecture in *Sasa*, revealed in this study, likely evolved as a consequence of adaptation to various environmental conditions, and particularly snow accumulation. The variation in branching architecture is strongly related to bud death and culm fragility, considering that the three types have a common development of the branching order established on a BO2 branch.

The boundary between the distributions of the Cr-type and those of the S- and M-types corresponds to 1 m of maximum snow accumulation on Hokkaido Island. As 1-m snow accumulation corresponds to soil freezing depth of −0.2 m (Higashi 1954), the Cr-type culms suffer from harsh environments both above and beneath the ground in regions with little snow accumulation. The culms are broken easily by their fragility and/or drying. Winter buds, which are formed near the ground level, can remain despite culm breaking and survive beneath leaf litter with high freezing tolerance (Konno 1977). Breaking culms can also save extra nutrients for subsequent shoot growth. It is an advantage of winter buds that the shoots grow rapidly in the following spring. Shorter culms among the other types can start early extending leaves. The branching system of the Cr-type is highly adapted to habitats with little snow accumulation.

The M-type culms are tough and flexible, as needed to survive heavy snow accumulation, with less risk of damage from extreme soil freezing. As the culms form spring buds to develop branches, the lateral buds in the dormant state are protected further under culm sheaths and leaf sheaths in winter. Many spring buds are formed at the upper node positions of tall culms and start growing on melting snow. The culms continue to develop higher branching orders for some years. The culms of this type also develop sylleptic branches. The M-type culms achieve the branching system adaptable to the heavy snow accumulation.

The branching system of the S-type shows a unique adaptation to the environment in its native habitat, which in contrast to those of the other two types can include highly variable amounts of snow accumulation. The S-type culms are distributed in transitional regions between heavy and little snow accumulation and between the habitats of the Cr- and M-types. The culms have both features of the Cr- and M-type ones with forming winter and spring buds. A large number of the winter buds die, irrespective of snow depth. As these buds and branching orders develop for only a few years, the S-type culms have smaller branching architecture than the M-type culms. The S-type culms are, however, superior to the M-type in some respects: the lateral buds are activated at lower positions of culms in S-type than in M-type, and they develop branches on the wider range. As the M-type culms adapt to heavy snow accumulation covering the culm height fully, they survive poorly when snow depth varies greatly. On the contrary, the S-type culms have a disadvantage in sites with little snow accumulation where the Cr-type occurs, because of the wider range of lateral buds and the ways for determination of their fate on the culms as mentioned above. The culms cannot maintain the large architecture even in the distribution area of the S-type. These were observed to grow much shorter than the Cr-type ones at the experimental plot E12 under 0-m snow depth. The boundary between the distributions of S-type and Cr-type corresponds to 1-m maximum snow depth in a colder region, Hokkaido Island, and 0.5-m maximum snow depth in warmer regions of the Japanese archipelago (S. Suzuki 1961). These imply that the S-type culms are superior to the Cr-type ones in the warmer environmental conditions.

The genera of Bambusoideae are widely distributed in the Japanese archipelago where snow accumulation varies greatly between the east (little snow accumulation) and west regions (heavy snow). The genus *Sasa* is most successful in the coldest northern district, Hokkaido Island, and the other genera in the warmer and more southern areas, and is the only genus in Bambusoideae that exhibits such wide morphological diversification between these two regions. The other genera, except for *Sasamorpha* are distributed in the both side regions and each genus displays a branching architecture without diversification. Thus, these genera are considered to evolve the branching systems with little influence of snow accumulation.

The genus *Sasamorpha* of the *Sasa* group is distributed only in the east side of the archipelago, and displays only one branching architecture. This architecture is similar to that of the M-type, despite its occurring in regions with little snow accumulation. *Sasamorpha* is predominant over the Cr-type in the warmer regions in the main Islands of the Japanese archipelago, though the genus has only a small distribution area in Hokkaido Island. The genus is distributed widely and represents the forest vegetation; *Fagus crenata–Sasamorpha purpurascens* association on the east in contrast to *F. crenata–S. kurilensis* association on the west (T. Suzuki 1952). This suggests that the Cr-type *Sasa* evolved the branching system adapting to little snow accumulation conditions under the colder environments.

## Concluding Remarks

The results of this study revealed that the branching architecture of the genus *Sasa* develops mainly through four endogenous processes and in response to environmental conditions, especially snow accumulation. Those processes include a distribution of lateral buds on a culm, internode length arrangement along a culm, determination of the fate of lateral buds and development of branching with culm fragility due to ageing. These processes correlated with each other within culms, as also reported in rice (Katayama 1944, 1951; Hoshikawa 1975; Yoshida 1981). It should be noted that the genus *Sasa* undergoes these correlations during the life span of the shoot to give rise to develop more complicated branching architecture. Such correlated development of branching systems is unique, compared with those of other plants, which have meristems only at shoot tips and in perennating buds, such as apple (*Malus domestica*) in which lateral branches occasionally develop depending on physiological conditions and/or environments (Lauri *et al.* 1997).

## Supporting Information

The following additional information is available in the online version of this article—

E1. Canopy species of the experimental plots in Hokkaido Island on Table 1.

E2. Schematic drawing of culms with node order coordinates (NDO).

plaa054_suppl_Supplementary_Material

## Data Availability

All data necessary to reproduce any analyses in this study have been provided as **Supporting Information Data**.

## Notes

**Form & Function. Chief Editor:** Kate McCulloh
